# Expression of calcium pumps is differentially regulated by histone deacetylase inhibitors and estrogen receptor alpha in breast cancer cells

**DOI:** 10.1186/s12885-018-4945-x

**Published:** 2018-10-23

**Authors:** Karolina Varga, Anna Hollósi, Katalin Pászty, Luca Hegedűs, Gergely Szakács, József Tímár, Béla Papp, Ágnes Enyedi, Rita Padányi

**Affiliations:** 1 2nd Department of Pathology, Semmelweis University, Budapest, Hungary; 2 Department of Biophysics and Radiation Biology, Semmelweis University, Budapest, Hungary; 3 Department of Thoracic Surgery, Ruhrlandklinik, University Clinic Essen, University Duisburg-Essen, Duisburg, Germany; 4 Institute of Enzymology, Research Centre for Natural Sciences, Hungarian Academy of Sciences, Budapest, Hungary; 5 Institute of Cancer Research, Medical University Vienna, Vienna, Austria; 6 U978, Institut National de la Santé et de la Recherche Médicale, and Université Paris-13, PRES Sorbonne Paris-Cité, Bobigny, France

**Keywords:** PMCA, Ca^2+^ signaling, Breast cancer cell lines, ER-α, 17β-estradiol, HDAC inhibitors, Valproate, SAHA

## Abstract

**Background:**

Remodeling of Ca^2+^ signaling is an important step in cancer progression, and altered expression of members of the Ca^2+^ signaling toolkit including the plasma membrane Ca^2+^ ATPases (PMCA proteins encoded by *ATP2B* genes) is common in tumors.

**Methods:**

In this study PMCAs were examined in breast cancer datasets and in a variety of breast cancer cell lines representing different subtypes. We investigated how estrogen receptor alpha (ER-α) and histone deacetylase (HDAC) inhibitors regulate the expression of these pumps.

**Results:**

Three distinct datasets displayed significantly lower *ATP2B4* mRNA expression in invasive breast cancer tissue samples compared to normal breast tissue, whereas the expression of *ATP2B1* and *ATP2B2* was not altered. Studying the protein expression profiles of Ca^2+^ pumps in a variety of breast cancer cell lines revealed low PMCA4b expression in the ER-α positive cells, and its marked upregulation upon HDAC inhibitor treatments. PMCA4b expression was also positively regulated by the ER-α pathway in MCF-7 cells that led to enhanced Ca^2+^ extrusion capacity in response to 17β-estradiol (E2) treatment. E2-induced PMCA4b expression was further augmented by HDAC inhibitors. Surprisingly, E2 did not affect the expression of PMCA4b in other ER-α positive cells ZR-75-1, T-47D and BT-474. These findings were in good accordance with ChIP-seq data analysis that revealed an ER-α binding site in the *ATP2B4* gene in MCF-7 cells but not in other ER-α positive tumor cells. In the triple negative cells PMCA4b expression was relatively high, and the effect of HDAC inhibitor treatment was less pronounced as compared to that of the ER-α positive cells. Although, the expression of PMCA4b was relatively high in the triple negative cells, a fraction of the protein was found in intracellular compartments that could interfere with the cellular function of the protein.

**Conclusions:**

Our results suggest that the expression of Ca^2+^ pumps is highly regulated in breast cancer cells in a subtype specific manner. Our results suggest that hormonal imbalances, epigenetic modifications and impaired protein trafficking could interfere with the expression and cellular function of PMCA4b in the course of breast cancer progression.

**Electronic supplementary material:**

The online version of this article (10.1186/s12885-018-4945-x) contains supplementary material, which is available to authorized users.

## Background

Breast cancer is the second most common cancer type worldwide, and is the major cause of cancer death among women according to the GLOBOCAN 2012 project [1]. Breast cancer is a very complex and heterogeneous disease that can be divided into several subtypes based on gene expression profiles and immunohistochemical markers. These subgroups also differ in their clinical outcomes, therapy responses and metastatic potential. Approximately 75% of breast cancers belong to the luminal A or B subtype, which are more differentiated breast tumors and express estrogen receptor alpha (ER-α) and/or progesterone receptor (PR). These cancers respond well to anti-estrogen therapy; however, recurrence is frequent. About 20% of breast cancers, that overexpress human epidermal growth factor receptor 2 (HER2) can be treated with targeted therapies against HER2. The most aggressive, triple negative (ER-, PR-, HER2-) basal and claudin-low subtypes still lack druggable targets; the only option for the systemic treatment of these tumors is chemotherapy [2, 3]. Therefore, breast cancer research aims to find new targets and new biomarkers that can predict therapy response or resistance. Recent studies suggest that targeting the PI3K/Akt/mTOR and FGFR or IGFR pathways, using PARP inhibitors, epigenetic modulators such as histone deacetylase (HDAC) inhibitors, or immunotherapies are the most promising pharmacological therapeutic opportunities [4–6]. HDAC inhibitors alone or in combination with other treatment options are under clinical investigation for the treatment of several solid malignancies, including breast cancer. These compounds exert their anticancer effects through inhibition of cell proliferation and induction of differentiation and cell death. Although the results are promising, especially in case of the combined therapies, the mechanisms of action are not completely understood [6–8].

The remodeling of cellular calcium (Ca^2+^) homeostasis is an important step in cancer progression, because Ca^2+^ signaling is linked either directly or indirectly to the main processes altered in tumorigenesis such as regulation of proliferation, cell survival, migration or invasion [9, 10]. The expression of several Ca^2+^ channels or pumps show characteristic changes during these processes, resulting in altered Ca^2+^ signal patterns affecting further downstream Ca^2+^-sensitive signaling pathways [9, 10]. Certain members of the transient receptor potential (TRP) family of the Ca^2+^ channels are frequently upregulated in various cancer types. Several studies have revealed the remodeling of store-operated Ca^2+^ entry (SOCE) and showed altered STIM and Orai protein expressions. Reduced expression of various forms of Ca^2+^ pumps was also observed in a variety of cancer types [9, 10]. The alteration of the sarco/endoplasmic reticulum Ca^2+^ ATPase 3 (SERCA3) – a SERCA-type Ca^2+^ pump – during tumorigenesis was confirmed in several different cancer types such as breast, lung, brain, colon, gastric carcinomas or leukemias, using either cancer cell lines or tissues [11–16].

Plasma membrane type Ca^2+^ pumps or Ca^2+^ ATPases (PMCAs) are responsible for the expulsion of Ca^2+^ from the cytosol into the extracellular space, to maintain a low intracellular Ca^2+^ concentration [17]. In mammals, four separate genes code for the major PMCA isoforms (*ATP2B1 ATP2B4* genes code PMCA1-PMCA4 proteins) and alternative RNA splicing can generate additional PMCA variants. The more than 20 splice variants show tissue- and cell type-specific expression and differ in their cellular localization and activity [18–20]. Since PMCA is a major regulator of Ca^2+^ signaling in many non-excitable cell types, it plays an essential role in the regulation of cell proliferation, differentiation and apoptosis, processes closely related to tumorigenesis [9, 20]. However, limited data are available on PMCAs related to cancer. Studies on colon cancer showed lower PMCA4 expression in tumors compared to normal tissue [21, 22], while PMCA1 was found to be downregulated in oral cancers [23]. Previous in vitro studies showed upregulated *ATP2B1* and *ATP2B2* mRNA and downregulated *ATP2B4* mRNA expression in some breast cancer cell lines [24, 25]. Moreover, high *ATP2B2* expression in breast cancer was found to be associated with specific tumor subtypes [26–28].

Previous studies have suggested that PMCAs can impact on Ca^2+^ signaling in an isoform specific manner [19]. Moreover, we found considerable PMCA4b upregulation during HDAC inhibitor treatment of MCF-7 breast cancer cells, and this effect was further enhanced by phorbol 12-myristate 13-acetate (PMA). The altered PMCA4b expression led to enhanced Ca^2+^ clearance, suggesting that the protein plays an important role in the Ca^2+^ homeostasis of MCF-7 cells. Moreover, immunohistochemical analysis of normal breast tissue showed high PMCA4 expression in breast ductal epithelial cells, suggesting that PMCA4 is an essential component of the Ca^2+^ signaling toolkit in the normal breast epithelium [29]. More recently, our group has found that HDAC inhibitors upregulated PMCA4b expression in melanoma cell lines [30]. In addition, we showed that inhibition of mutant B-Raf enhanced PMCA4b expression in *BRAF* mutant melanoma cells, and that PMCA4b abundance (induced either by overexpression or drug treatments) was coupled with decreased migration and metastatic activity of *BRAF* mutant melanoma cells [31]. These observations also highlight the importance of PMCA4b in the development and progression of these tumor types.

In this study we investigated the expression of the *ATP2B* genes in publicly available breast cancer gene expression datasets and studied the modulation of the expression of various Ca^2+^ pumps at the protein level by HDAC inhibitor and/or 17β-estradiol (E2) treatments in a variety of breast cancer cell lines. The examined cell line panel represents the non-tumorigenic breast epithelium, the luminal A, luminal B and HER2 expressing, as well as the triple negative, basal subtype breast tumors (see Additional file 1: Table S1) [2, 3, 32–34]. Publicly available data [35] revealed significantly lower *ATP2B4* mRNA expression in breast carcinomas when compared to normal breast tissue. Protein levels of the PMCA and SERCA isoforms showed high variability among the cell lines, and distinct regulatory mechanisms of PMCA expression were observed upon drug treatments or ER-α activation, depending on tumor subtype.

## Methods

### Cell culture

The MCF-10A, MCF-7, ZR-75-1, BT-474, AU-565, SK-BR-3 and Hs578T cell lines were obtained from the American Type Culture Collection (ATCC). The T-47D, MDA-MB-468, BT-549 and MDA-MB-231 cell lines were obtained from NCI Development Therapeutics Program (DCTD Tumor Repository, National Cancer Institute at Frederick, MD). Stocks of frozen viable cells were generated immediately after one or two passages, and low passage number cells were used for all experiments. All cell lines were tested for mycoplasma infection with MycoSensor PCR Assay kit (Agilent Technologies) and only mycoplasma free cells were used for the experiments. The MCF-7, GCaMP2-MCF-7, SK-BR-3 and Hs578T cells were cultured in DMEM supplemented with 10% FBS (Gibco, Thermo Scientific), 100 U/ml penicillin, 100 μg/ml streptomycin and 2 mM glutamine. The T-47D, ZR-75-1, BT-474, AU-565, MDA-MB-468, BT-549, MDA-MB-231 and GCaMP2-MDA-MB-231 cells were cultured in RPMI 1640 supplemented with 10% FBS (Gibco, Thermo Scientific), 100 U/ml penicillin, 100 μg/ml streptomycin and 2 mM glutamine. The MCF-10A cell line was cultured in MEGM according to the instructions of ATCC. Cells were incubated at 37 °C and 5% CO_2_ in a humidified atmosphere.

### Stable GCaMP2 expressing cell lines

The GCaMP2-MCF-7 cell line was established previously as described in [29]. For further studies a similar GCaMP2-MDA-MB-231 cell line was generated using the Sleeping Beauty transposon system, as described earlier [29].

### Reagents and treatments

Valproic acid sodium salt (VPA; Sigma-Aldrich) was dissolved in sterile distilled water, membrane filtered and stored at − 20 °C. Suberoylanilide hydroxamic acid (SAHA; Sigma-Aldrich), 17β-estradiol (E2; Sigma-Aldrich) and fulvestrant (ICI 182,780; Sigma-Aldrich) stock solutions were made in DMSO and stored at − 20 °C. The final DMSO concentration did not exceed 0.01% in all experiments, DMSO vehicle was included in controls and did not interfere with the experiments.

For HDAC inhibitor treatments, exponentially growing cells were seeded in 6-well plates for Western blotting or in 8-well chambers (Nunc Lab-Tek II chambered coverglass, Thermo Scientific) for immunocytochemistry and Ca^2+^ signal measurements, and incubated for 3 days until cultures reached ~ 80% confluency. Culture medium was then replaced by fresh medium, and VPA or SAHA was added from concentrated stock solutions. During SAHA treatment the medium was replaced daily. After 4 days of treatment, protein expressions were analyzed by Western blotting or by immunocytochemistry, or Ca^2+^ signal measurements were performed. In the case of E2 treatments, cells were incubated in E2-free culture medium (DMEM w/o phenol red or RPMI 1640 w/o phenol red, supplemented with 10% charcoal-stripped FBS (Gibco, Thermo Scientific), 100 U/ml penicillin, 100 μg/ml streptomycin and 2 mM glutamine). For Western blotting or Ca^2+^ signal measurements cells were seeded in 6-well plates or in 8-well chambers (Nunc Lab-Tek II chambered coverglass, Thermo Scientific) respectively, in normal growth medium, and after 1 day medium was replaced by E2-free medium, and cells were incubated for 2 days until cultures reached ~ 80% confluency. E2-free medium was then renewed, and cells were treated with 1 nM E2 with or without 100 nM fulvestrant and the indicated amount of HDAC inhibitors. After 4 days of treatment, protein expressions were analyzed or Ca^2+^ signal measurements were performed.

### Western blot analysis

Total protein extraction from the cells was obtained by precipitation with 6% TCA, and Western blotting was performed as described previously [36]. Equal amounts of total cellular protein were loaded on polyacrylamide gels (7.5, 10 or 15% depending on the examined protein), electrophoresed and electroblotted onto PVDF membranes (Bio-Rad). Immunostainings were performed with the following primary antibodies: mouse monoclonal anti-*pan* PMCA (5F10), rabbit polyclonal anti-PMCA1 (NR1), rabbit polyclonal anti-PMCA2 (NR2), rabbit polyclonal anti-PMCA3 (NR3), mouse monoclonal anti-PMCA4 (JA9), and mouse monoclonal anti-PMCA4b (JA3) described in [37, 38], mouse monoclonal anti-SERCA2 antibody (IID8; Sigma-Aldrich), mouse monoclonal anti-SERCA3 (PL/IM430; [39]), mouse monoclonal anti-β-actin (AC-15; Sigma-Aldrich), mouse monoclonal anti-ER-α (6F11; Invitrogen), rabbit polyclonal anti-ER-β (Invitrogen) and rabbit polyclonal anti-acetyl-histone H3 (Lys9/Lys14) (Cell Signaling). Signals of the secondary, HRP-conjugated anti-mouse or anti-rabbit antibodies (Jackson ImmunoResearch) were detected using the Pierce ECL Western Blotting Substrate (Thermo Scientific) and luminography on CL-XPosure Film (Thermo Scientific) or Amersham Hyperfilm ECL (GE Healthcare) films. Densitometric analyses were carried out using the ImageJ software v1.51j8, and data were processed with the Prism 4 software v4.01 (GraphPad Software) and expressed as means ± SEM.

### Immunocytochemistry

The procedure of immunocytochemical staining was performed as described previously [29] with a mouse monoclonal anti-PMCA4b (JA3) primary antibody [37] and an Alexa Fluor 488-conjugated anti-mouse IgG secondary antibody (Invitrogen). Images were taken by a Zeiss LSM710 confocal laser scanning microscope equipped with a Plan-Apochromat 63×/1.40 oil immersion objective and Zeiss ZEN software. Images of control and treated wells from the same cell line were taken with the same microscope settings.

### Ca^2+^ signal measurement

GCaMP2-MCF-7 and GCaMP2-MDA-MB-231 cell lines were used for Ca^2+^ signal measurements after VPA treatment. Cells were treated with 4 mM VPA for 4 days. In the case of E2 treatments, GCaMP2-MCF-7 cells were preincubated in E2-free DMEM (DMEM w/o phenol red, supplemented with 10% charcoal-stripped FBS (Gibco), 100 U/ml penicillin, 100 μg/ml streptomycin and 2 mM glutamine) for 2 days and then treated with 1 nM E2 for 4 days. Immediately before the Ca^2+^ signal measurement, medium was replaced by HBSS supplemented with with 0.9 mM MgCl_2_, 2 mM CaCl_2_ and 20 mM HEPES; pH 7.4. Ca^2^ influx was triggered by the addition of 2 μM A23187 (Sigma-Aldrich). Fluorescent signal of the GCaMP2 Ca^2+^ sensor was detected by confocal imaging with a Zeiss LSM710 confocal laser scanning microscope and Plan-Apochromat 63×/1.40 oil immersion objective. Time lapse images were recorded every 0.3 s at room temperature with the Zeiss ZEN software. Data were analyzed with the ImageJ software v1.51j8 and Prism 4 software v4.01 (GraphPad Software). The relative fluorescence intensities were calculated as F/F_0_ (where F_0_ was the average initial fluorescence) and are expressed as means ±95% CI. Statistical significance was calculated by t-test, *** means *P* < 0.001, ** means *P* < 0.01, * means *P* < 0.05, n.s. means not significant.

### Analysis of publicly available ChIP-seq data

ChIP-seq data analysis was performed using the Cistrome Data Browser [40, 41]. Used raw data from the Gene Expression Omnibus (GEO) [42, 43] are: GSM798428 (ER-α Chip-seq data in T-47D cells, this data sample is in the GEO data series GSE32222 [44]), GSM798424 (ER-α Chip-seq data in MCF-7 cells, this data sample is in the GEO data series GSE32222 [44]), GSM986089 (ER-α Chip-seq data in ZR-75-1 cells, this data sample is in the GEO data series GSE40129 [45]), GSM986063 (ER-α Chip-seq data in MCF-7 cells, this data sample is in the GEO data series GSE40129 [45]), GSM2040043 (ER-α Chip-seq data in MCF-7 cells [46]), GSM2467223 (ER-α Chip-seq data in MCF-7 cells [Dzida et al., unpublished]). Chip-seq data were visualized using the UCSC Genome Browser [47, 48] on Human Dec. 2013 (GRCh38/hg38) Assembly.

## Results

### *ATP2B4* gene expression is downregulated in breast carcinomas

**Fig. 1:**
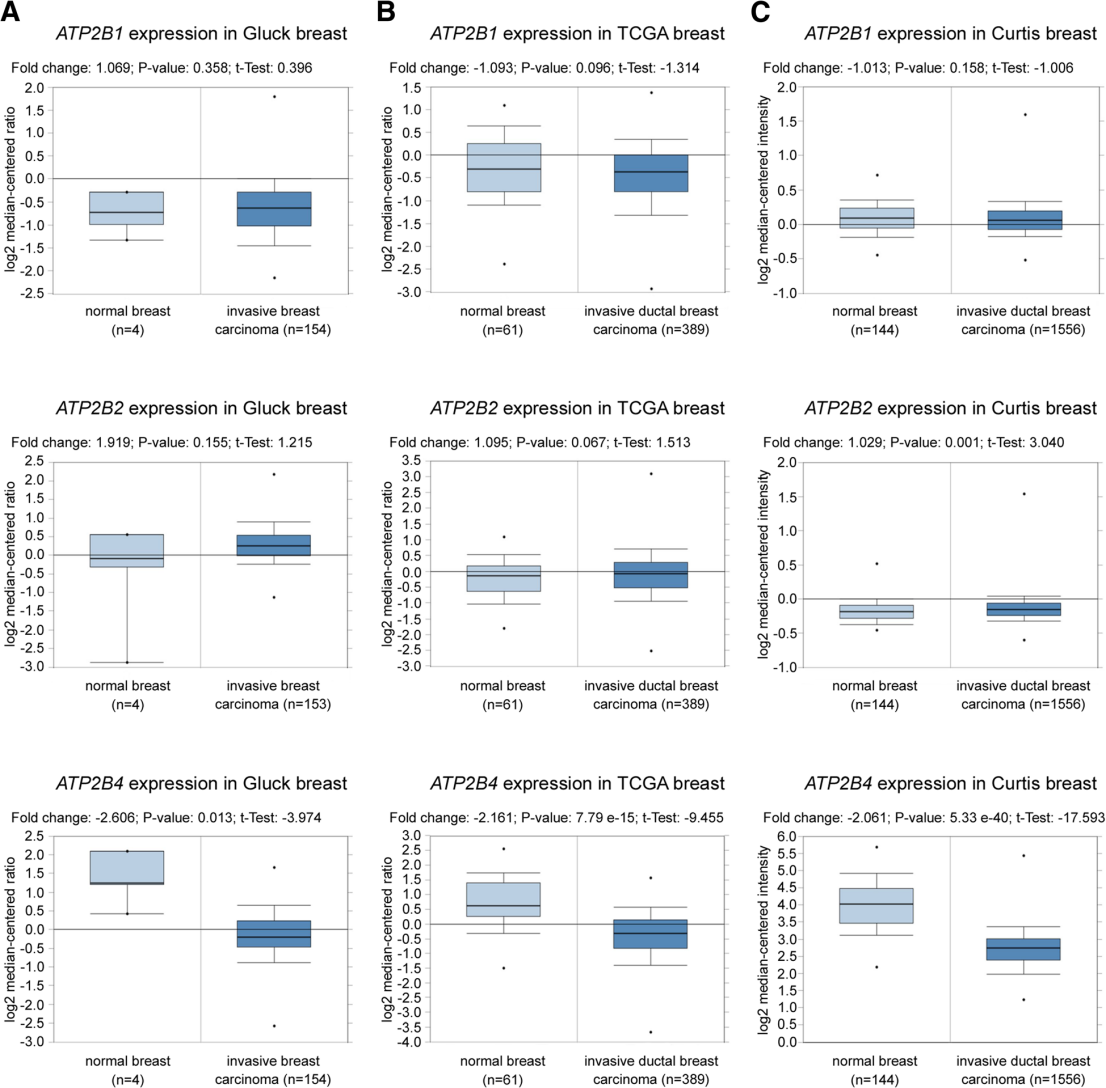
*ATP2B4* gene expression is downregulated in breast carcinomas compared to normal breast tissue. *ATP2B1, ATP2B2* and *ATP2B4* gene expression data were obtained from the Oncomine database [35]. **a:** Relative gene expression of normal (*n* = 4) vs. invasive breast carcinoma (*n* = 154) tissues from the „Gluck breast” study [76]. *ATP2B1*: fold change: 1.069; *P*-value: 0.358; t-test: 0.396. *ATP2B2*: fold change: 1.919; *P*-value: 0.155; t-test: 1.215. *ATP2B4*: fold change: − 2.606; *P*-value: 0.013; t-test: − 3.974. **b:** Relative gene expression of normal (*n* = 61) vs. invasive ductal breast carcinoma (*n* = 389) tissues from „TCGA breast” study (2011). *ATP2B1*: fold change: − 1.093; *P*-value: 0.096; t-test: -1.314. *ATP2B2*: fold change: 1.095; *P*-value: 0.067; t-test: 1.513. *ATP2B4*: fold change: − 2.161; *P*-value: 7.79 e-15; t-test: − 9.455 **c:** Relative gene expression of normal (*n* = 144) vs. invasive ductal breast carcinoma (*n* = 1556) tissues from „Curtis breast” study [77]. *ATP2B1*: fold change: − 1.013; *P*-value: 0.158; t-test: -1.006. *ATP2B2*: fold change: 1.029; *P*-value: 0.001; t-test: 3.040. *ATP2B4*: fold change: − 2.061; *P*-value: 5.33 e-40; t-test: − 17.593

In our previous study immunohistochemical staining of breast tissue indicated the presence of PMCA4 (*ATP2B4*) protein in normal breast ductal epithelium [29]. Here, we evaluated the expression of the *ATP2B* family members in breast cancer tissues analyzing data from three individual gene expression datasets using the Oncomine database [35]. We found that the expression of the *ATP2B4* gene was downregulated in invasive breast carcinoma or invasive ductal breast carcinoma samples compared to normal breast tissues (Fig. 1). In contrast, the expression of the *ATP2B1* and *ATP2B2* genes was not altered in breast cancer tissues. Next we studied Ca^2+^ pump expressions in vitro in a set of breast cancer cell lines representing all breast tumor types, at the protein level.

### Different breast cancer subtypes have different Ca^2+^ pump expression patterns

**Fig. 2:**
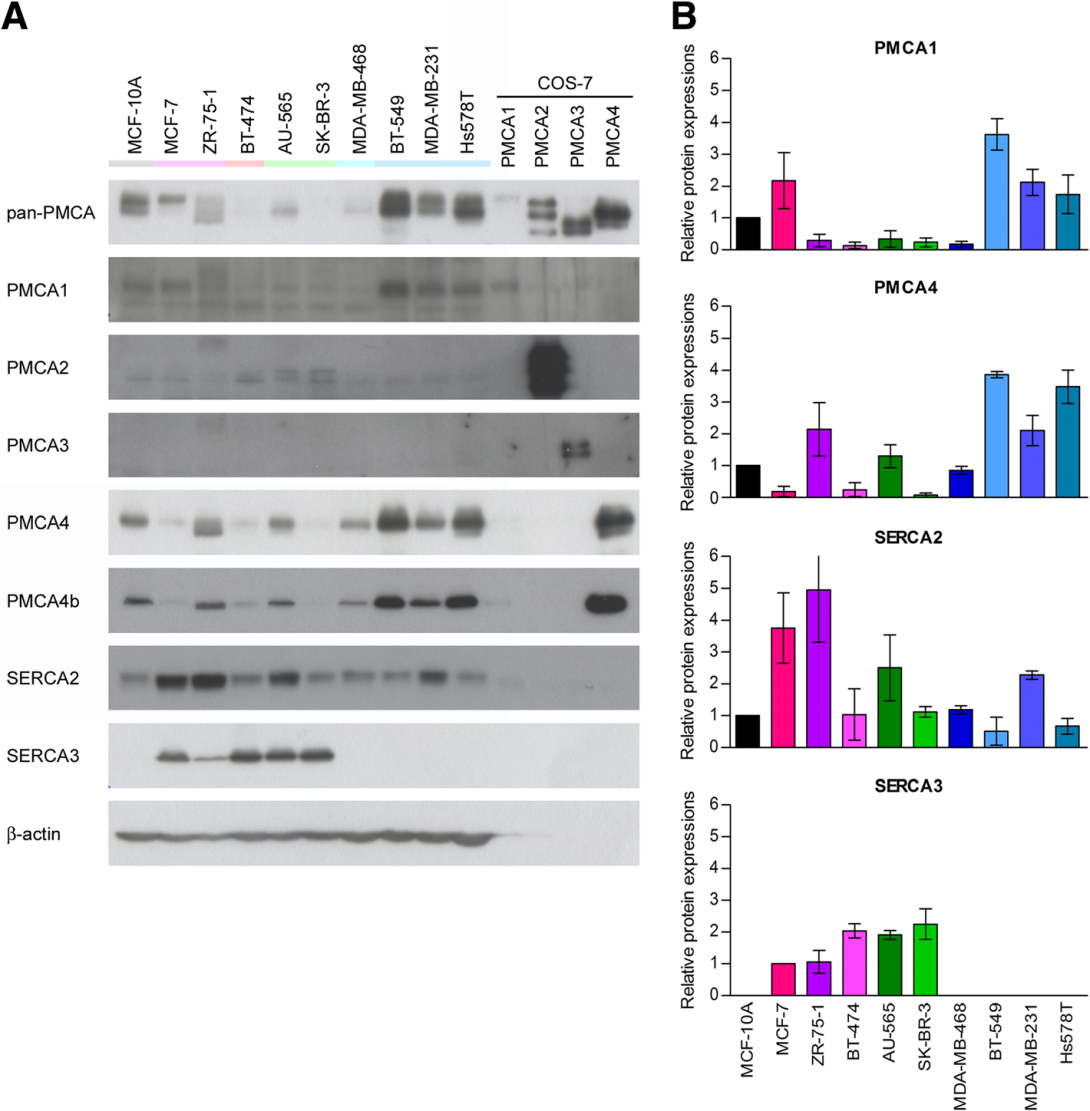
Diverse Ca^2+^ pump expressions were found in breast tumor cell lines representing different subtypes. Basal protein expression of PMCA and SERCA isoforms was analyzed by Western blotting in different breast cancer cell lines. **a:** Equal amount (30 μg) of total cell protein lysates of confluent cell cultures were immunostained with isoform specific antibodies: anti-pan-PMCA (5F10), anti-PMCA1 (NR1), anti-PMCA2 (NR2), anti-PMCA3 (NR3), anti-PMCA4 (JA9), anti-PMCA4b (JA3), anti-SERCA2 (IID8) and anti-SERCA3 (PL/IM 430). β-actin served as a loading control. Microsomal membrane preparations isolated from COS-7 cells were used as isoform-specific positive controls: PMCA1: 1 μg membrane protein from untransfected cells; PMCA2: a mixed sample of 0.1–0.1 μg membrane protein from cells transfected with rPMCA2a, rPMCA2b, hPMCA2wb constructs; PMCA3: a mixed sample of 0.2–0.2 μg membrane protein from cells transfected with rPMCA3a, rPMCA3b constructs; PMCA4: a mixed sample of 0.2–0.2 μg membrane protein from cells transfected with hPMCA4a, hPMCA4xb constructs. **b:** Relative protein expression of PMCA1, PMCA4, SERCA2 and SERCA3 isoforms. Densitometric values were normalized to the respective β-actin levels and compared to the protein expression of the non-tumorigenic MCF-10A cells in the case of PMCA1, PMCA4 and SERCA2, or to the protein expression of MCF-7 in the case of SERCA3. Bars represent mean ± SEM from two independent experiments

We examined the expression of PMCA and SERCA proteins in a variety of breast carcinoma cell lines (see Additional file 1: Table S1) by Western blotting, using isoform specific antibodies (Fig. 2). Low PMCA4b expression was found in the luminal subtype and HER2 overexpressing cells, whereas the triple negative, basal cancer cells, representing a more aggressive breast cancer subtype showed high PMCA4b and PMCA1 expression levels. PMCA2 specific staining was very low in all cell types, and no PMCA3 was found in any of the examined cell lines. SERCA3 was expressed in all luminal subtypes and HER2 overexpressing cells, while it was totally absent in the triple negative cells, including also the non-tumorigenic MCF-10A line. This correlates well with previous observations showing that triple negative ductal breast carcinomas have reduced SERCA3 expression [15]. The housekeeping SERCA2 protein was present in all cell types at a relatively high abundance.

### Expression of Ca^2+^ pumps is modulated by HDAC inhibition in a cell type specific manner

**Fig. 3:**
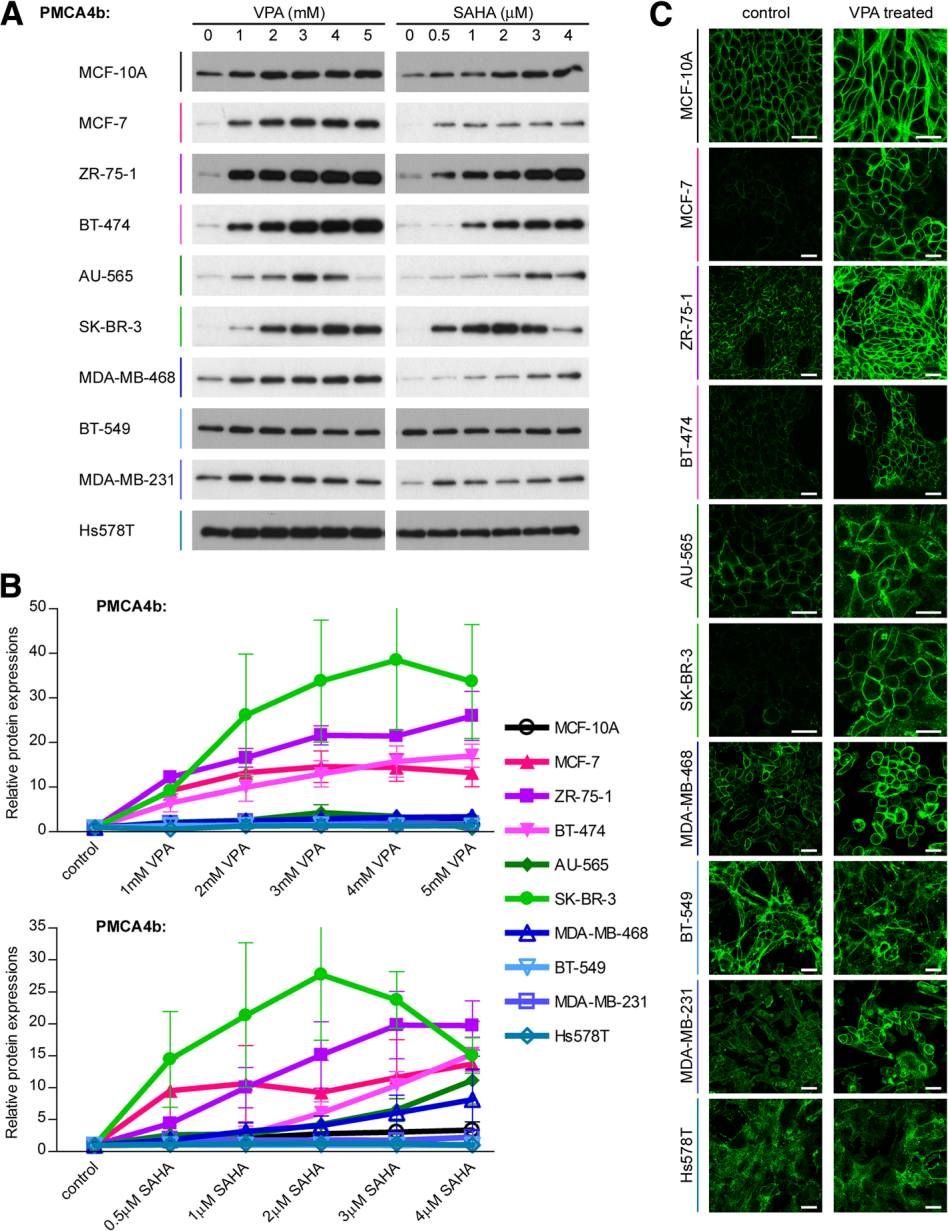
PMCA4b expression is selectively regulated during HDAC inhibitor treatments in breast cancer cell lines. Effects of valproate (VPA) and suberoylanilide hydroxamic acid (SAHA) treatments on PMCA4b protein expression in different breast cancer cell lines. **a:** Cells were treated with increasing concentrations of VPA or SAHA as indicated for 4 days, and PMCA4b protein expression from total cell lysates (15 μg protein per sample) was analyzed by Western blotting with JA3 antibody. **b:** Relative PMCA4b expression after a 4 day VPA or SAHA treatment. Densitometric values were normalized to the respective β-actin loading control levels and expressed as fold increase over the untreated controls. Bars represent mean ± SEM from two to four independent experiments. **c:** Subcellular localization of the PMCA4b protein in VPA-treated cells. MCF-7, ZR-75-1, BT-474 and BT-549 cells were treated with 2 mM VPA for 4 days. MCF-10A, AU-565, SK-BR-3, MDA-MB-468, MDA-MB-231 and Hs578T cells were treated with 4 mM VPA for 4 days. After the treatments, every cell line was immunostained with an anti-PMCA4b antibody (JA3). Images were taken by confocal microscopy. Scale bar: 30 μm

Previously we reported that HDAC inhibitor and PMA treatments caused pronounced elevation in the mRNA and protein expression levels of PMCA4b in MCF-7 cells [29]. Here we treated different breast cancer cell lines and the non-tumorigenic MCF-10A cells with the FDA-approved HDAC inhibitor valproate (VPA) or suberoylanilide hydroxamic acid (SAHA; Vorinostat), which are currently tested in clinical trials for breast cancer treatment [6–8]. We found a substantial upregulation of PMCA4b expression in the luminal subtype cell lines (MCF-7, ZR-75-1, BT-474) and in the HER2 overexpressing SK-BR-3 cell line. The upregulation was less pronounced in all triple negative, basal type cancer cells, MDA-MB-468, BT-549, MDA-MB-231, Hs578T and in the non-tumorigenic MCF-10A cell line and in the HER2 positive AU-565 cells. It is important to emphasize that the basal type cells that included the non-tumorigenic MCF-10A cells, as well, had already high PMCA4b levels without any treatments (Fig. 3a-b). PMCA4b expression decreased after treatments with high concentration VPA or SAHA in case of AU-565 and SK-BR-3 cells, suggesting that these cell lines are more sensitive to prolonged treatments with high dosage HDAC inhibitors that may induce cell death. Elevation of the PMCA1 isoform was induced only in ZR-75-1 and BT-549 cells, and SERCA2 and SERCA3 expressions changed only slightly upon HDAC inhibitor treatments (see Additional file 2: Figure S1).

Previous studies have shown that HDACs are overexpressed in ER-α positive breast tumors, and HDAC1, HDAC3 and HDAC6 protein expressions correlate with ER-α expression [49, 50]. Therefore, we examined the level of histone H3 acetylation after HDAC inhibitor treatments in the investigated cell lines. We found considerable upregulation of histone H3 acetylation in the ER-α positive breast cancer cells in response to HDAC inhibition that correlated well with PMCA4b expression (see Additional file 3: Figure S2). These results suggest that alteration in histone acetylation levels is involved in the regulation of PMCA4b expression. However, we could not detect such a close correlation between PMCA4b expression and histone H3 acetylation in the case of the HER2 positive and triple negative cell lines. PMCA4b was found to be upregulated in the HER2 positive SK-BR-3 cell line without considerable enhancement of histone H3 acetylation, whereas in the triple negative BT-549 cells, HDAC inhibitors significantly elevated histone H3 acetylation without any effect on PMCA4b expression.

### Impaired PMCA4b localization in triple negative cells suggest loss of cellular function

**Fig. 4:**
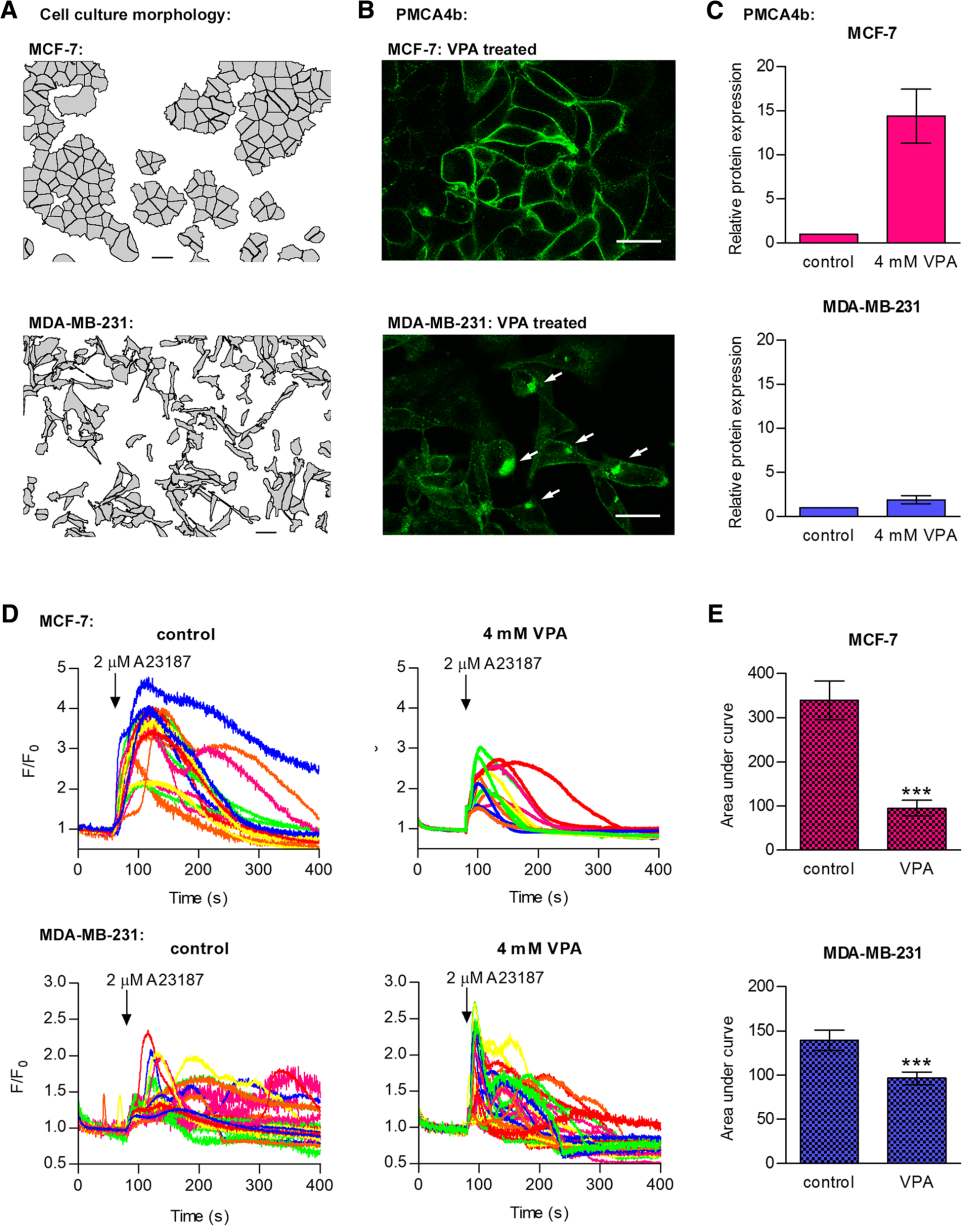
PMCA4b localization and function is impaired in the triple negative MDA-MB-231 cell line. **a**: Cell culture morphology analysis of the MCF-7 and MDA-MB-231 cell lines. Pictures were taken with a phase contrast microscope. Gray cell masks were made using the ImageJ software v1.51j8 to emphasize the morphology of the cell cultures and cell-cell contacts. Scale bar: 30 μm. **b**: Subcellular localization of the PMCA4b protein in VPA-treated MCF-7 and MDA-MB-231 cells. MCF-7 cells were treated with 2 mM VPA, MDA-MB-231 cell were treated with 4 mM VPA for 4 days and immunostained with an anti-PMCA4b antibody (JA3). Images were taken by confocal microscopy. White arrows indicate PMCA4b protein in intracellular compartments. Scale bar: 30 μm. **c**: Relative PMCA4b expression in MCF-7 and MDA-MB-231 cells after a 4 day VPA treatment. Densitometric values of Western blots were normalized to the respective β-actin loading control levels and expressed as fold increase over the untreated controls. Bars represent mean ± SEM from two to four independent experiments. **d**: Ca^2+^ signal measurements in VPA-treated GCaMP2-MCF-7 and GCaMP2-MDA-MB-231 cells. Cells were treated with 4 mM VPA for 4 days. Before the measurement, culture medium was replaced by HBSS supplemented with 2 mM Ca^2+^. Ca^2+^ influx was triggered by 2 μM Ca^2+^ ionophore A23187, and fluorescent signal of the GCaMP2 Ca^2+^ sensor was followed by confocal imaging. F/F_0_ values represent individual cells (*n* = 14–32) from a representative experiment. **e**: Area under curve of the A23187-induced Ca^2+^ transients. Bar graphs are means ± SEM of the individual cells (*n* = 14–32). Significance between control and VPA-treated cells is denoted by *** (*P* < 0.001); two-tailed unpaired t-test

A previous study demonstrated that PMCA4b can reach the plasma membrane only in fully confluent HeLa cell culture [51]. Similarly to these cells, PMCA4b was located to the plasma membrane in all luminal type and HER2 positive cells displaying epithelial type cell morphology with intact cell-cell contact sites (Figs. 3c and 4a-b). In order to test the effect of the enhanced PMCA4b expression on Ca^2+^ signaling, GCaMP2 expressing MCF-7 cells were treated with VPA and challenged with the Ca^2+^ ionophore A23187. Using this protocol we found that the enhanced plasma membrane PMCA4b expression was accompanied with an enhanced Ca^2+^ extrusion capacity of the cells, decreasing the amplitude and duration of the Ca^2+^ signal (Fig. 4d-e). The basal type MDA-MB-231 cells represent a more aggressive type of breast tumors that display an elongated cell shape and a scattered pattern even at high density. The lack of well-defined cell-cell contact sites can be the reason why a large amount of PMCA4b seems to be located in intracellular compartments in these cells (Figs. 3c and 4a-b). Previously we showed that in HeLa cells the level of confluency was important in the regulation of plasma membrane localization of PMCA4b. In non-confluent HeLa cell cultures a substantial portion of PMCA4b was also located intracellularly [51]. In MDA-MB-231 cells VPA treatment did not change the expression of the pump significantly (Fig. 4c) in good correlation with the much less pronounced effect of the treatment on the A23187 induced Ca^2+^ transient (Fig. 4d-e). These experiments suggest that in the basal type cells a considerable amount of PMCA4b is located in intracellular compartments where it probably cannot perform its plasma membrane-associated function [51].

### 17β-estradiol enhances PMCA4b protein expression in MCF-7 cells

**Fig. 5:**
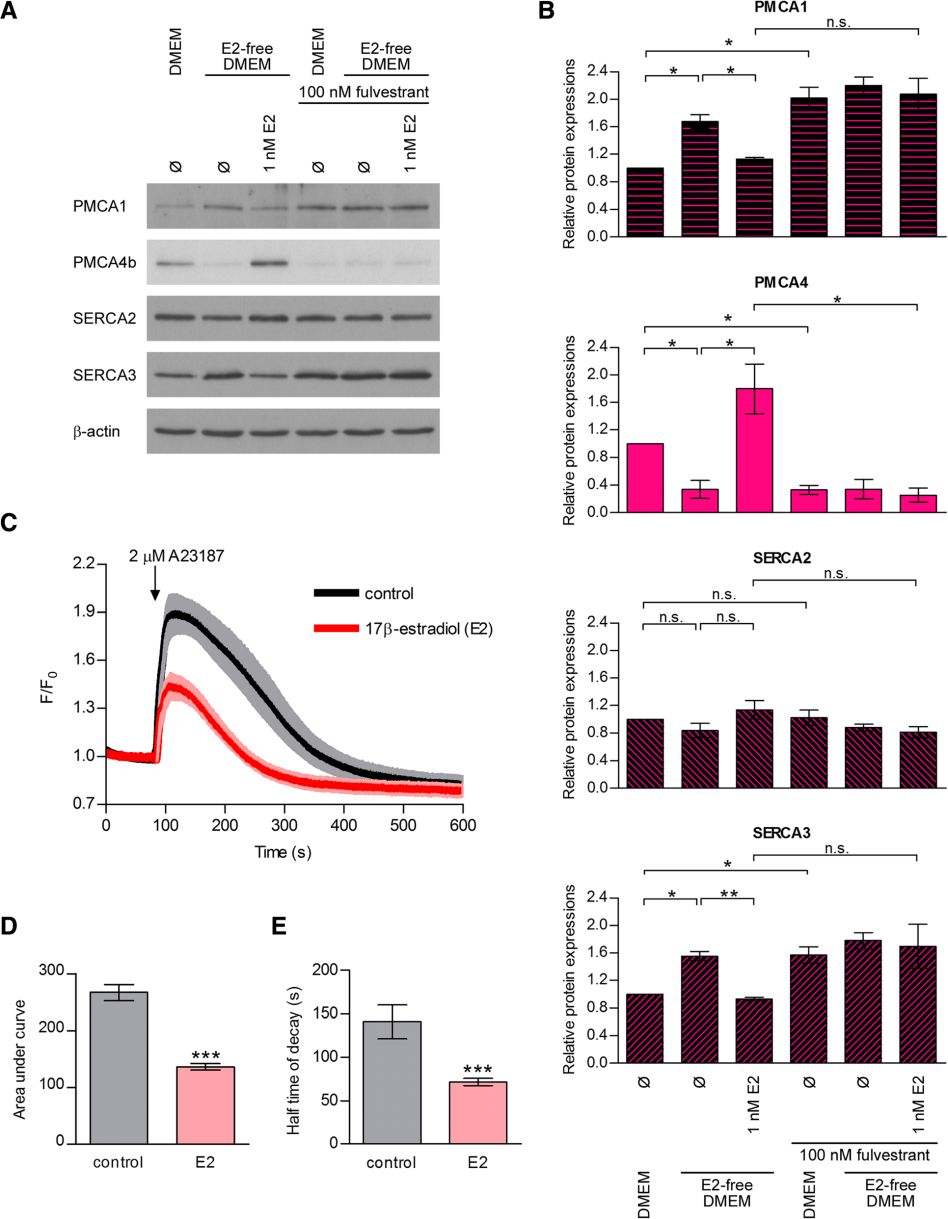
17β-estradiol enhances PMCA4b protein expression and activity in MCF-7 cells. 17β-estradiol (E2)-dependence of PMCA4b protein expression and activity. **a**: MCF-7 cells were cultured in DMEM or in E2-free DMEM and treated with 1 nM E2 ± 100 nM fulvestrant (fulv.) for 4 days as indicated. Equal amount (30 μg) of total cell lysates were analyzed by Western blotting with isoform specific antibodies: anti-PMCA1 (NR1), anti-PMCA4b (JA3), anti-SERCA2 (IID8) and anti-SERCA3 (PL/IM 430). β-actin served as a loading control. **b**: Relative protein expression of PMCA1, PMCA4b, SERCA2 and SERCA3 isoforms. Densitometric values were normalized to the respective β-actin levels and compared to the protein expression of untreated cells cultured in DMEM. Bars represent mean ± SEM from three independent experiments. Significance is denoted by * (*P* < 0.05) or ** (*P* < 0.01); two-tailed paired t-test. **c**: Ca^2+^ signal measurement in E2-treated GCaMP2-MCF-7 cells. Cells were cultured in E2-free DMEM and treated with 1 nM E2 for 4 days. Before the measurement, culture medium was replaced by HBSS supplemented with 2 mM Ca^2+^. Ca^2+^ influx was triggered by 2 μM Ca^2+^ ionophore A23187, and fluorescent signal of the GCaMP2 Ca^2+^ sensor was followed by confocal imaging. Data represent means of normalized values (F/F_0_) ± 95% CI of 41 control and 59 E2-treated cells collected from three independent experiments. **d**: Area under curve of the A23187-induced Ca^2+^ transients. Bar graphs are means ± SEM of the individual cells taken from three independent experiments. Significance between control and E2-treated cells is denoted by *** (*P* < 0.001); two-tailed unpaired t-test. **e**: Half time of decay of the A23187-induced Ca^2+^ transients. Bar graphs are means ± SEM of the individual cells taken from three independent experiments. Significance between control and E2-treated cells is denoted by *** (*P* < 0.001); two-tailed unpaired t-test

Previous observations have shown that PMCA isoform switch is a characteristic feature of the normal mammary epithelial cell physiology during pregnancy and lactation, suggesting that PMCAs are under hormonal control in the breast [20, 52, 53]. To gain insight into the regulation of PMCA expression in hormone receptor positive breast cancer cells, we examined if estrogen receptor alpha (ER-α) regulated PMCA expression in MCF-7 cells (Fig. 5a-b). Because normal complete growth medium contains estrogen, and phenol-red that also displays estrogenic activity [54], cells were cultured in a phenol-red free medium supplemented with charcoal-stripped estrogen-free FBS before and during 17β-estradiol (E2) treatments. We found a much lower PMCA4b protein expression when cells were cultured in this nominally hormone-free medium than in the normal complete DMEM used in our previous experiments. PMCA4b expression highly increased when hormone-free medium was supplemented with E2. Furthermore, the pure ER-α antagonist fulvestrant (ICI 182,780) completely reversed the E2-induced PMCA4b upregulation, indicating that the effect of E2 was specific. It is important to note that the expression of PMCA1 and SERCA3 slightly decreased upon E2 treatment suggesting a mild compensatory mechanism for the PMCA4b upregulation.

We then examined if the E2-induced changes in the Ca^2+^ pump levels affected the Ca^2+^ signal in MCF-7 cells. We performed Ca^2+^ signal measurements using a previously generated MCF-7 cell line that expresses the genetically encoded Ca^2+^ indicator GCaMP2 [29]. We preincubated GCaMP2-MCF-7 cells in E2-free medium and then treated the cells with E2 or DMSO vehicle. Ca^2+^ signals were initiated by the addition of the Ca^2+^ ionophore A23187, and changes in the fluorescence of the GCaMP2 sensor were monitored by confocal imaging (Fig. 5c and Additional file 4: Figure S3). We found that in E2-treated cells the Ca^2+^ peak was significantly smaller (Fig. 5d), and the intracellular Ca^2+^ concentration returned to the baseline level significantly faster than in the untreated cells (Fig. 5e). These results suggest an enhanced Ca^2+^ extrusion capacity of cells as a result of a net increase in PMCA expression in response to the E2-induced ER-α activity.

### HDAC inhibitor treatment potentiates the ER-α-induced PMCA4b expression in MCF-7 cells

**Fig. 6:**
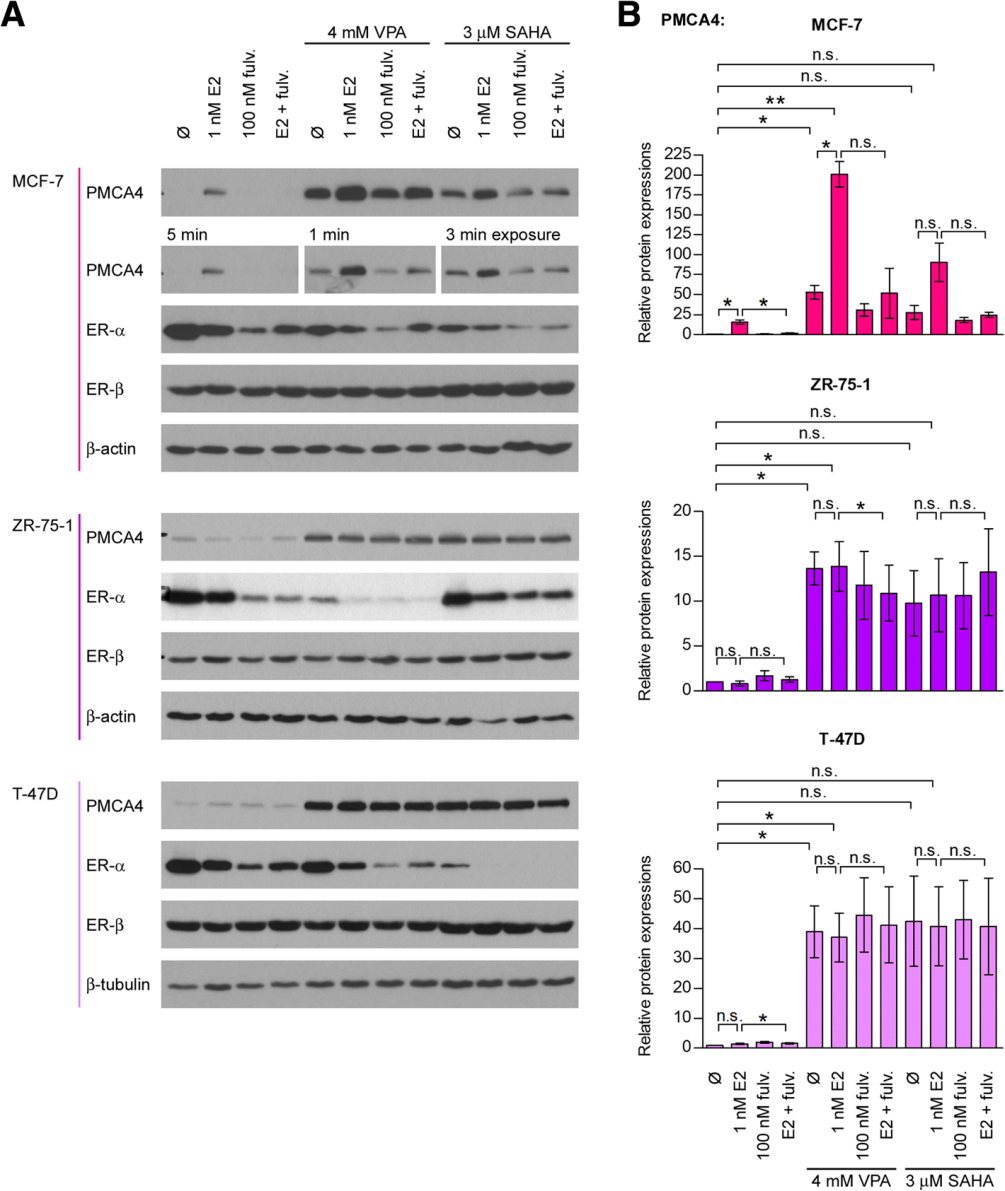
17β-estradiol enhances PMCA4 expression specifically in MCF-7 and further enhances the HDAC inhibitor induced upregulation. Effects of 17β-estradiol (E2) ± HDAC inhibitor treatments on PMCA4 protein expression in ER-α positive breast cancer cell lines. **a**: MCF-7, ZR-75-1 and T-47D cells were cultured in E2-free culture medium and treated with 1 nM E2 ± 100 nM fulvestrant (fulv.) ± 4 mM VPA or 3 μM SAHA for 4 days as indicated. Equal amount (30 μg) of total cell lysates were analyzed by Western blotting with anti-PMCA4 (JA9), anti-ER-α and anti-ER-β antibodies. β-actin served as a loading control. **b**: Relative PMCA4 protein expression in the examined cell lines. Densitometric values were normalized to the respective β-actin levels and expressed as fold increase over the untreated controls. Bars represent mean ± SEM from three independent experiments. Significance is denoted by * (*P* < 0.05) or ** (*P* < 0.01); two-tailed paired t-test

Next, we combined E2 treatment with VPA or SAHA in MCF-7 cells cultured in E2-free medium, and found that combination of the two drugs further enhanced PMCA4b expression (Fig. 6). It is worth mentioning, however, that the HDAC inhibitors alone - in the absence of E2 - also increased PMCA4b abundance. The ER-α antagonist fulvestrant fully reversed the E2-specific fraction of the PMCA4b upregulation, whereas it did not alter the estrogen-independent effects of HDAC inhibitors, indicating that additional regulatory mechanisms are also involved.

### Regulation of PMCA4 by ER-α is specific to the MCF-7 cell line

We also examined the effect of E2 on PMCA4 expression in other ER-α positive cell lines (T-47D, ZR-75-1, BT-474) and in the ER-α negative MDA-MB-231 (Fig. 6 and Additional file 5: Figure S4). E2 treatment did not influence PMCA4 expression in any of these cells suggesting that the regulation of PMCA4 level by ER-α was specific to MCF-7 cells. We showed, however, that ER-α was present in all of the ER-α positive cells at a comparable level, and its expression decreased substantially after fulvestrant and HDAC inhibitor treatments, as expected [49]. Thus, all ER-α positive cell lines reacted to fulvestrant, but PMCA4 expression changed only in MCF-7 cells during the manipulation of ER-α activity. This is an important finding since MCF-7 cells are often used as a model system for studying ER-α positive breast tumor types.

**Fig. 7:**
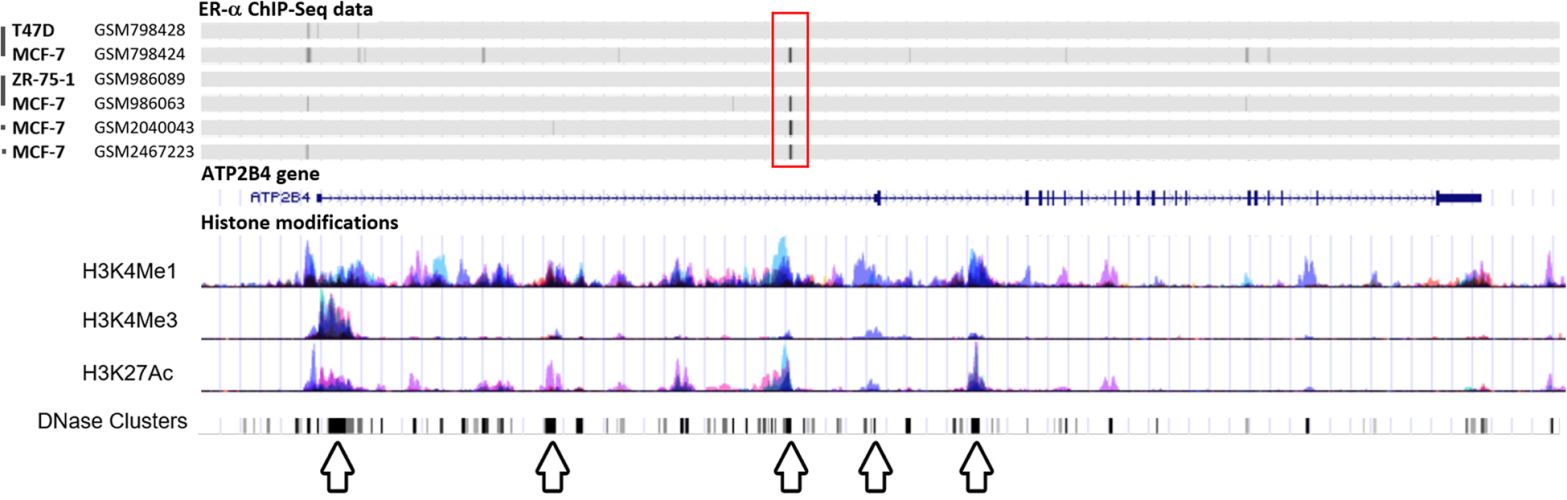
*ATP2B4* gene has an ER-α bindig site in the intron 1 in MCF-7 cells. The human *ATP2B4* locus (chromosome 1, 1q32.1) in the UCSC Genome Browser (Human Dec. 2013 (GRCh38/hg38) Assembly) [47, 48]. The RefSeq *ATP2B4* transcript contains the untranslated exon 1 and 22 protein coding exons. Arrows mark transcriptionally important sites according to histone methylation and acetylation patterns. Binding site of ER-α (obtained from ChIP-seq data of Cistrome database [40, 41]) is marked with red rectangle. ChIP-Seq data were sourced from GSM2467223 [Dzida et al., unpublished], GSM2040043 [46], GSM986063 and GSM986089 from series GSE40129 [45], GSM798424 and GSM798428 from series GSE32222 [44]

To further study the role of ER-α in the regulation of PMCA4 expression, we analyzed data from chromatin immunoprecipitation sequencing (ChIP-seq) experiments of ER-α binding to the *ATP2B4* gene locus in ER-α positive breast cancer cells. We used the Cistrome Data Browser [40, 41] to identify ER-α binding sites, and compared data from different ChIP-seq libraries. Analysis of four independent ChIP-seq data samples on MCF-7 cells identified an active ER-α binding site in the intron 1 of the *ATP2B4* gene (marked with red rectangle in Fig. 7). Within this region histone modification marks (H3K4Me1, H3K4Me3 methylation and H3K27Ac acetylation) were also observed indicating the presence of regulatory binding sites. Next, we compared ChIP-seq data of MCF-7, ZR-75-1 and T-47D cells from experiments containing either MCF-7 and ZR-75-1 or MCF-7 and T-47D data pairs and found that ER-α bound to the *ATP2B4* gene locus in MCF-7 cells, but not in the other two ER-α positive cell lines ZR-75-1 and T-47D (Fig. 7). These observations are in good correlation with our experimental results demonstrating that among the examined ER-α positive cell lines ER-α regulates PMCA4 expression only in MCF-7 cells.

## Discussion

Altered expression of the members of the Ca^2+^ signaling toolkit frequently occurs during cancer progression. However, the literature does not contain much published research on Ca^2+^ pumps in tumors. In breast cancer cells upregulated *ATP2B1*, *ATP2B2* mRNA and downregulated *ATP2B4* mRNA expressions were described previously [24, 25]. Using bioinformatic tools we found that the expression of the *ATP2B4* gene is significantly lower in invasive breast cancer tissue samples than in normal breast tissue, while that of the *ATP2B1* and *ATP2B2* genes did not show considerable differences. However, in vitro examination of Ca^2+^ pump protein levels in different breast cancer cell lines showed various expression levels of PMCA4b and other PMCA isoforms. The function of isoform 2 in the normal physiology of breast epithelium during lactation is well documented [52, 55–57]. More recent studies examined the role of PMCA2 in breast cancer [26–28]. Comparing different breast cancer subtypes we found that *ATP2B2* mRNA expression was elevated in basal type cancers, and correlated positively with survival [28]. Controversially, elevated *ATP2B2* expression was found to be associated with poor clinical outcome in breast cancer in another study [27]. PMCA2 also regulates HER2 signaling in HER2 positive breast cancer cells [26]. HER2 was found to interact with PMCA2 in specific membrane domains where the low local Ca^2+^ concentration supports sustained HER2 signaling and tumor growth [26]. In our in vitro experiments PMCA2 protein expression was low in all examined breast cancer cell lines, with PMCA1 and PMCA4b being the main isoforms, although both were expressed at variable levels. We found considerable SERCA3 protein (encoded by the ATP2A3 gene) expression in all the examined luminal and HER2 overexpressing cell lines, while it was not detectable in the triple negative, basal subtype cells including MCF-10A. This observation is in accordance with former studies that showed loss of SERCA3 expression during tumorigenesis, and decreased SERCA3 expression in triple negative breast cancers [15].

HDAC inhibitors such as VPA or SAHA are currently being developed for various types of malignancies, including breast cancer [6, 58], and are already in clinical use for peripheral T cell lymphomas [59, 60]. In this work VPA and SAHA, two structurally different HDAC inhibitors increased PMCA4b expression in breast cancer cells, and enhanced PMCA4b expression induced by E2 in MCF-7 cells. Unlike that of E2, the effect of VPA and SAHA on PMCA4b expression was not inhibited by fulvestrant. These observations taken together indicate that PMCA4b expression in MCF-7 cells is controlled by ER-α-, as well as by HDAC-dependent chromatin remodeling. MCF-7 cells constitute a unique in vitro model for the study of the E2-dependent control of PMCA4b expression and of the cross-talk between sex hormone and Ca^2+^ signaling pathways.

The effect of the HDAC inhibitors was much less pronounced in the triple negative cells, where the initial PMCA4b expression was higher. We found that in the triple negative MDA-MB-231 cells a considerable amount of PMCA4b protein was located in intracellular compartments, suggesting that PMCA4b cannot perform its plasma membrane-associated function, similarly to that seen in non-confluent HeLa cells [51]. It is important to note that mislocalization of other proteins, including those with tumor suppressor function has been reported in association with cancer development and metastasis [61]. It has been shown that ER-α positive breast tumors express HDAC proteins at higher amounts [49, 50], and that the antiproliferative effect of HDAC inhibitors is also more potent in the ER-α positive breast cancer cell lines [62]. Therefore, it is not surprising that in our experiments HDAC inhibitors showed higher effect on PMCA4b expression in the luminal type cell lines. However, histone acetylation levels and PMCA4b upregulation did not correlate tightly in the HER2 overexpressing or basal cell lines. *ATP2A3* mRNA was found to be overexpressed after HDAC inhibitor treatment both in the MCF-7 and MDA-MB-231 cell lines [63] but we could not find any significant changes in SERCA3 protein expression in response to VPA or SAHA. Several studies aim to elucidate the mechanism of HDAC inhibition to develop more potent strategies for cancer treatment. Some of these studies showed that HDAC inhibitors induced expression of ER-α in triple negative breast cancer cell lines or reversed hormone resistance of the ER-α positive cells [49, 64, 65]. These ER-α negative or tamoxifen/aromatase inhibitor-resistant breast tumors could be sensitized to anti-estrogen therapies by HDAC inhibitors that induce the expression of the epigenetically repressed ER-α receptor. Clinical trials have also been performed with HDAC inhibitors alone or in combination with tamoxifen [64, 66]. Although, some results were promising, controversial data were also reported in other studies, in which HDAC inhibitors did not induce ER-α expression in the triple negative breast cancer cells [6, 67]. In our experiments ER-α protein expression in MDA-MB-231 cells was also not affected by VPA or SAHA.

As we already mentioned above, here we describe for the first time that activation of the ER-α pathway increases PMCA4b protein expression in MCF-7 cells. However, the expression of the protein did not change in the other examined ER-α positive breast cancer cell lines ZR-75-1, T-47D, BT-474 or in the ER-α negative MDA-MB-231 cells. Our results are in line with those studies that report complex regulation of the ER-α-induced transcription of target genes [34, 64, 68]. The in-depth analysis of a gene chip assay revealed upregulation of the *ATP2B4* gene in response to E2 stimulation of MCF-7 cells [68]. Interestingly, *ATP2B4* was also upregulated by E2 in MDA-MB-231 cells stably expressing exogenous ER-α [68]. Stender et al. discussed that the ability of ER-α to regulate gene expression in different cell lines in a different way depended on many factors, such as varying transcription factor expression and activity, different chromatin structure or epigenetic modifications [68]. Hilborn et al. examined the effect of E2 on the expression of hydroxysteroid 17β-dehydrogenase (*HSD17B*) 1 and 2 [69]. The protein products of *HSD17B1* and *HSD17B2* play an important role in controlling E2 activity. In their experiments *HSD17B2* expression was upregulated after a 7-days E2 treatment in MCF-7 but not in T-47D and ZR-75-1 cells [69]. This result further supports the idea that different ER-α positive breast cancer cell lines use different regulatory pathways in response to ER-α activation, and are in accordance with our results indicating that among the ER-α positive cell lines only MCF-7 is E2-responsive in terms of elevation of PMCA4b expression.

The G protein-coupled estrogen receptor 1 (GPER/GRP30) plays a role in non-genomic ER-α signaling [64]. Besides mediating a wide range of cellular processes, such as activation of the cAMP, ERK1/2 and PI3K pathways or intracellular Ca^2+^ mobilization [70], GPER/GRP30 can affect metastasis progression, as it was reported to regulate the cell-matrix adhesion of MCF-7 cells through the ERK1/2-calpain pathway [71]. Previously, PMCA4 was found to form a protein complex with the GPER/GRP30, in which GPER/GPR30 inhibits PMCA4 activity and PMCA4 also affects GPER/GRP30 function [72]. GPER/GPR30 was described as a E2-binding receptor, but it can be activated both by E2 and the pure ER-α antagonist fulvestrant [70]. In our experimental system, fulvestrant completely inhibited the E2-induced PMCA4 upregulation, suggesting that GPER/GPR30 is not involved in this process. Further studies are needed to clarify the exact mechanisms of E2 on the regulation of Ca^2+^ pump expression.

E2-dependent signaling is central for the physiological regulation of normal breast epithelial cell function, growth, differentiation and survival, and estrogen directs the growth also of ER-α positive breast cancer cells [73]. Antiestrogen therapy by agents such as fulvestrant is therefore a mainstay of ER-α positive breast cancer therapy [74]. Data presented in this work show that ER signaling, and its pharmacological modulation modify the Ca^2+^ homeostasis of cells via the regulation of PMCA4b expression. It has been shown earlier that, due to its slow activation/inactivation kinetics, the presence or the absence of PMCA4b determines the spatiotemporal characteristics of SOCE-type Ca^2+^ transients and oscillations [19], which in turn affect cell survival, motility and proliferation. In addition, PMCA4b expression has been shown earlier in normal ductal mammary epithelial cells in situ [29]. The demonstration of the E2 dependency of PMCA4b expression is therefore an interesting new aspect of mammary epithelial cell differentiation present in ER-α positive breast cancer cells, that constitutes a previously unknown mechanism of cross-talk between E2- and Ca^2+^-dependent signaling. This may be exploited in the future, for example for devising new combination anticancer therapies whereby E2- and Ca^2+^-dependent signaling mechanisms are simultaneously targeted in breast cancer cells.

It is also well known that the expression of various PMCA isoforms is tightly regulated in breast tissue, especially during pregnancy and lactation [55–57], further highlighting the physiological importance of our findings. The main PMCA isoform is PMCA4b in the developing rat mammary tissue, and its expression is increased during pregnancy. However, PMCA4b protein shows a major rapid downregulation after parturition when PMCA2b is significantly upregulated, and plays an essential role in transporting Ca^2+^ into the milk during lactation [52]. The changes in PMCA4b abundance coincided with gradually increasing serum E2 levels during pregnancy that drastically drop prior to parturition [75]. While further evidence is needed to prove a direct interaction between the ER and PMCA4, these results suggest that the effect of E2 on PMCA4 expression is physiologically relevant. Moreover, the altered PMCA4 expression and the reshaped Ca^2+^ signal pattern in breast tumor cells suggest that the protein might have an important role in neoplasia.

## Conclusions

In this study we found that the expression of Ca^2+^ pumps is differentially regulated by epigenetic drugs and estradiol in breast cancer cell lines having different genetic backgrounds. HDAC inhibitor treatments induced pronounced upregulation of the PMCA4b protein in ER-α positive luminal type cells. Importantly, we show for the first time, that the ER-α pathway upregulates PMCA4b expression specifically in the MCF-7 cell line. Identification of an active ER-α binding site in the *ATP2B4* gene in MCF-7 cells further supports our experimental observation. Our work also shows that changes in Ca^2+^ pump expression levels shape calcium extrusion and thus the intracellular Ca^2+^ signals that can further affect several downstream signaling pathways.

## Additional files

Additional file 1: **Table S1**. Breast cancer cell lines and their characteristics used in this study [2, 3, 32–34]. (PDF 185 kb)

Additional file 2: **Figure S1**. Effects of VPA and SAHA treatments on Ca^2+^ pump expressions in different breast cancer cell lines. **A:** Cells were treated with increasing concentration of VPA or SAHA as indicated for 4 days, and protein expressions from total cell lysates (15 μg protein per sample) were analyzed by Western blotting with isoform specific antibodies: anti-PMCA1 (NR1), anti-SERCA2 (IID8) and anti-SERCA3 (PL/IM 430). **B:** Relative PMCA1, SERCA2 and SERCA3 protein expressions after a 4 day VPA or SAHA treatment. Densitometric values were normalized to the respective β-actin loading control levels and expressed as fold increase over the untreated controls. Bars represent mean ± SEM from two to four independent experiments. (TIF 5113 kb)

Additional file 3: **Figure S2**. Effects of VPA and SAHA treatments on PMCA4b protein expression and histone H3 acetylation level in different breast cancer cell lines. **A:** Cells were treated with 4 mM VPA or 3 μM SAHA for 4 days, and protein expressions from total cell lysates (30 μg protein per sample) were analyzed by Western blotting with JA9 and anti-acetyl-histone H3 antibodies. **B:** Relative protein expressions from a representative experiment. Densitometric values were normalized to the respective β-actin loading control levels, and expressed as fold increase over the untreated controls in the case of each cell line. (TIF 990 kb)

Additional file 4: **Figure S3**. Ca^2+^ signal measurement in E2-treated GCaMP2-MCF-7 cells. Cells were cultured in E2-free DMEM and treated with 1 nM E2 for 4 days. Before the measurement, culture medium was replaced by HBSS supplemented with 2 mM Ca^2+^. Ca^2+^ influx was triggered by 2 μM Ca^2+^ ionophore A23187, and fluorescent signal of the GCaMP2 Ca^2+^ sensor was followed by confocal imaging. F/F_0_ values represent individual cells (41 control and 59 E2-treated cells) collected from three independent experiments. (TIF 602 kb)

Additional file 5: **Figure S4**. Effects of 17β-estradiol (E2) ± HDAC inhibitor treatments on PMCA4 protein expression in the ER-α positive BT-474 and in the ER-α negative MDA-MB-231 breast cancer cell lines. **A:** BT-474 and MDA-MB-231 cells were cultured in E2-free culture medium and treated with 1 nM E2 ± 100 nM fulvestrant (fulv.) ± 4 mM VPA or 3 μM SAHA for 4 days as indicated. Equal amounts (30 μg) of total cell lysates were analyzed by Western blotting using the anti-PMCA4 (JA9), anti-ER-α and anti-ER-β antibodies. β-actin served as a loading control. **B:** Relative PMCA4 protein expression in the examined cell lines. Densitometric values were normalized to the respective β-actin levels and expressed as fold increase over untreated controls. Bars represent mean ± SEM from three independent experiments. (TIF 915 kb)

## Abbreviations

AP-1: activator protein 1
ATCC: American Type Culture Collection
ChIP-seq: chromatin immunoprecipitation sequencing
DMEM: Dulbecco’s Modified Eagle Medium
DMSO: dimethyl sulfoxide
E2: 17β-estradiol
ERE: estrogen response element
ER-α: estrogen receptor alpha
FBS: fetal bovine serum
FDA: US Food and Drug Administration
GEO: Gene Expression Omnibus
GPER/GRP30: G protein-coupled estrogen receptor 1
HBSS: Hank’s balanced salt solution
HDAC: histone deacetylase
HEPES: 4-(2-Hydroxyethyl) piperazine-1-ethanesulfonic acid
HER2: human epidermal growth factor receptor 2
*HSD17B*: hydroxysteroid 17β-dehydrogenase
MEGM: Mammary Epithelial Cell Growth Medium
PMA: phorbol 12-myristate 13-acetate
PMCA (*ATP2B*): plasma membrane Ca^2+^ ATPase
PR: progesteron receptor
RPMI 1640: Roswell Park Memorial Institute 1640 Medium
SAHA: suberoylanilide hydroxamic acid (Vorinostat)
SERCA (*ATP2A*): sarco/endoplasmic reticulum Ca^2+^ ATPase
SOCE: store-operated Ca^2+^ entry
Sp1: specificity protein 1
TCA: trichloroacetic acid
TRP: transient receptor potential channels
VPA: valproate
