# Supplementary material for: Expression of calcium pumps is differentially regulated by histone deacetylase inhibitors and estrogen receptor alpha in breast cancer cells
Source: BMC Cancer. 2018 Oct 23;18:1029. doi: 10.1186/s12885-018-4945-x (PMC6199715; doi:10.1186/s12885-018-4945-x)
Supplement: Supplementary file 1 — Table S1. Breast cancer cell lines and their characteristics used in this study [2, 3, 32–34]. (PDF 185 kb) [file 12885_2018_4945_MOESM1_ESM.pdf]

Table S1: Breast cancer cell lines and their characteristics used in this study [2, 3, 30-32].

Cell line	MCF-10A	MCF-7	T-47D	ZR-75-1	BT-474	AU-565	SK-BR-3	MDA-MB-468	BT-549	MDA-MB-231	Hs578T
Tumor type	non-tumorigenic breast epithelial cell line	invasive ductal carcinoma	invasive ductal carcinoma	invasive ductal carcinoma	invasive ductal carcinoma	metastatic adeno-carcinoma	metastatic adeno-carcinoma	metastatic adeno-carcinoma	invasive ductal carcinoma	metastatic adeno-carcinoma	invasive ductal carcinoma
Intrinsic subtype		Luminal A			Luminal B	HER2		Basal	Basal/Claudin-low		
TNBC subtype								BL1 (basal-like 1)	M (mesenchymal)	MSL (me-senchymal stem-like)	MSL (me-senchymal stem-like)
IHC markers: ER	-	+	+	+	+/-	-	-	-	-	-	-
IHC markers: PR	-	+	+	+/-	+	-	-	-	-	-	-
IHC markers: HER2	-	-	-	-	+	+	+	-	-	-	-
Histologic grade		Low			Intermedi-ate/Low	High		High	High		
Prognosis		Excelent			Intermedi-ate/Bad	Bad		Bad	Bad		
Other characteristics		Ki67 low; better differentiated tumors; endocrine responsive, often chemotherapy responsive			Ki67 high; usually endocrine responsive, variable to chemotherapy	Ki67 high; usually responsive to HER2-targeted therapies, chemotherapy responsive		EGFR+ and/or cytokeratin 5/6+; Ki67 high; agressive and poorly differentiated tumors; often chemotherapy responsive	E-cadherin, claudin-3, -4 and -7 low; Ki67 low; agressive and poorly differentiated tumors; intermediate response to chemotherapy		
Mutations	CDKN2A/B	CDKN2A, PIK3CA	PIK3CA, TP53	PTEN	PIK3CA, TP53	TP53	TP53	PTEN, RB1, SMAD4, TP53	PTEN, RB1, TP53	BRAF, CDKN2A, KRAS, TP53	HRAS, CDKN2A, TP53
