# Supplementary figures and images for: Expression of calcium pumps is differentially regulated by histone deacetylase inhibitors and estrogen receptor alpha in breast cancer cells
Source: BMC Cancer. 2018 Oct 23;18:1029. doi: 10.1186/s12885-018-4945-x (PMC6199715; doi:10.1186/s12885-018-4945-x)

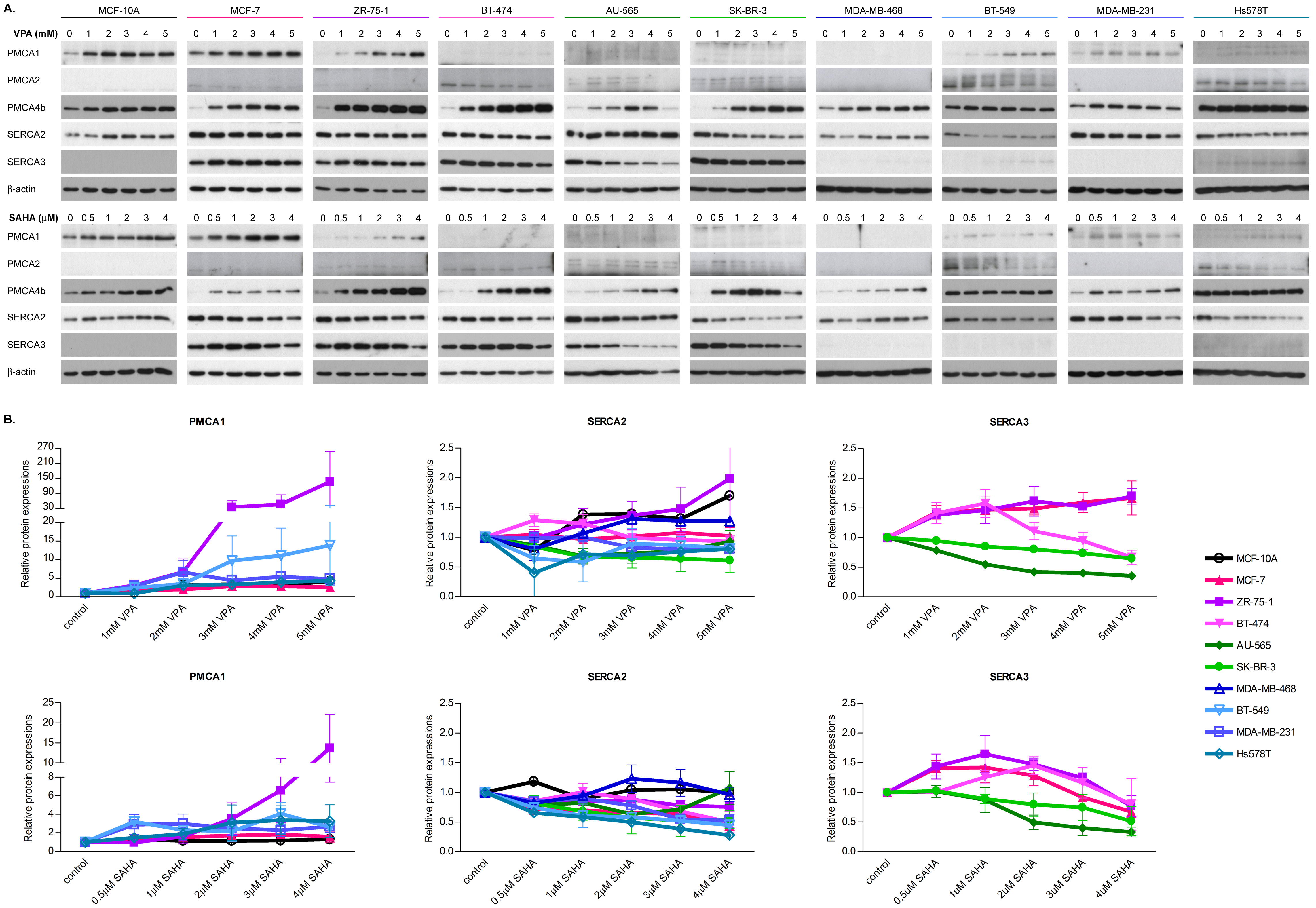

Supplement: Supplementary file 2 — Figure S1. Effects of VPA and SAHA treatments on Ca2+ pump expressions in different breast cancer cell lines. A: Cells were treated with increasing concentration of VPA or SAHA as indicated for 4 days, and protein expressions from total cell lysates (15 μg protein per sample) were analyzed by Western blotting with isoform specific antibodies: anti-PMCA1 (NR1), anti-SERCA2 (IID8) and anti-SERCA3 (PL/IM 430). B: Relative PMCA1, SERCA2 and SERCA3 protein expressions after a 4 day VPA or SAHA treatment. Densitometric values were normalized to the respective β-actin loading control levels and expressed as fold increase over the untreated controls. Bars represent mean ± SEM from two to four independent experiments. (TIF 5113 kb) [file 12885_2018_4945_MOESM2_ESM.tif]

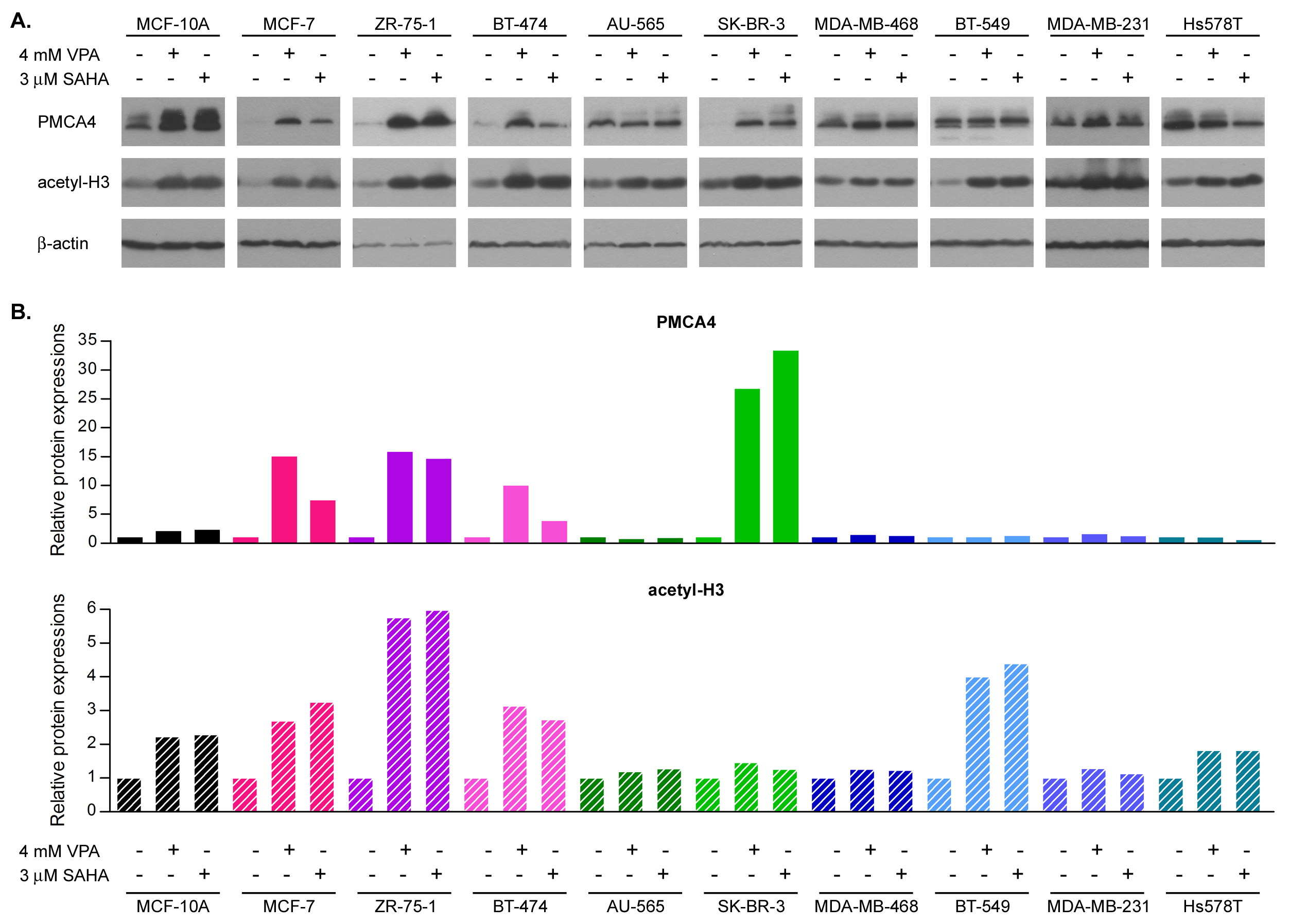

Supplement: Supplementary file 3 — Figure S2. Effects of VPA and SAHA treatments on PMCA4b protein expression and histone H3 acetylation level in different breast cancer cell lines. A: Cells were treated with 4 mM VPA or 3 μM SAHA for 4 days, and protein expressions from total cell lysates (30 μg protein per sample) were analyzed by Western blotting with JA9 and anti-acetyl-histone H3 antibodies. B: Relative protein expressions from a representative experiment. Densitometric values were normalized to the respective β-actin loading control levels, and expressed as fold increase over the untreated controls in the case of each cell line. (TIF 990 kb) [file 12885_2018_4945_MOESM3_ESM.tif]

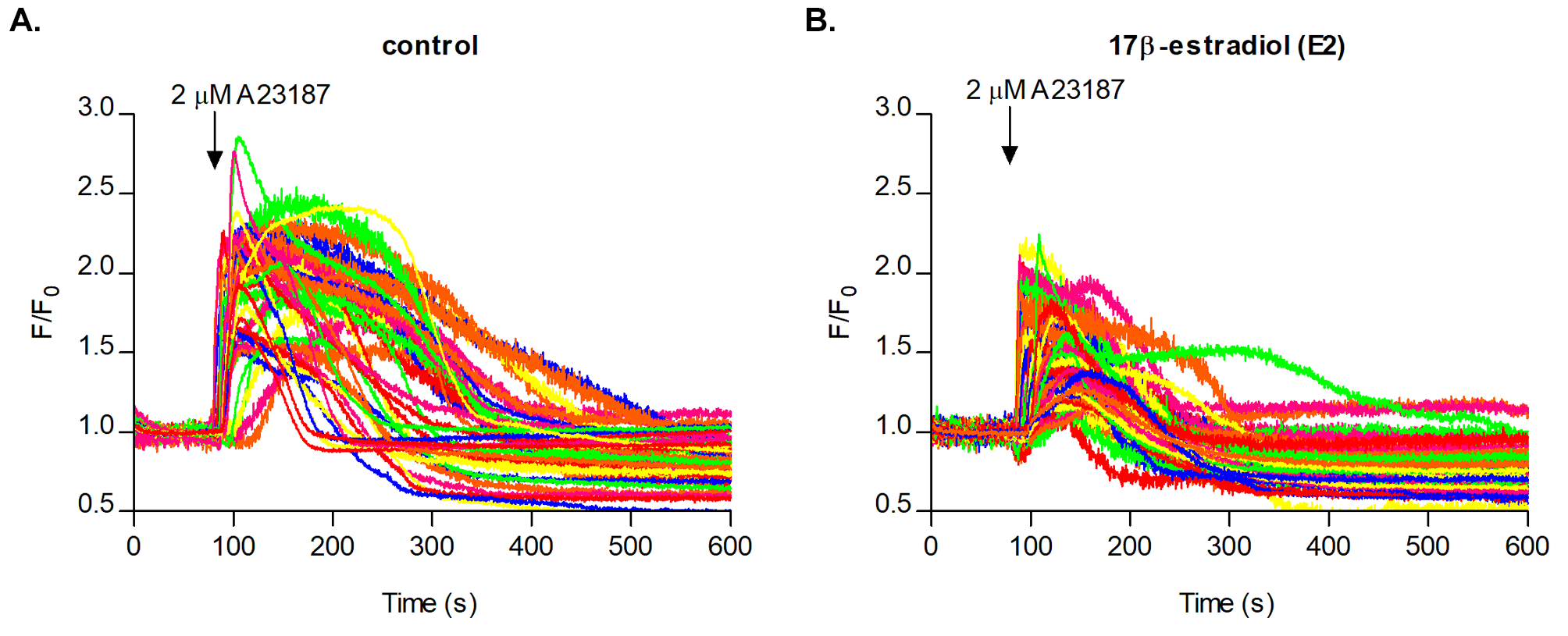

Supplement: Supplementary file 4 — Figure S3. Ca2+ signal measurement in E2-treated GCaMP2-MCF-7 cells. Cells were cultured in E2-free DMEM and treated with 1 nM E2 for 4 days. Before the measurement, culture medium was replaced by HBSS supplemented with 2 mM Ca2+. Ca2+ influx was triggered by 2 μM Ca2+ ionophore A23187, and fluorescent signal of the GCaMP2 Ca2+ sensor was followed by confocal imaging. F/F0 values represent individual cells (41 control and 59 E2-treated cells) collected from three independent experiments. (TIF 602 kb) [file 12885_2018_4945_MOESM4_ESM.tif]

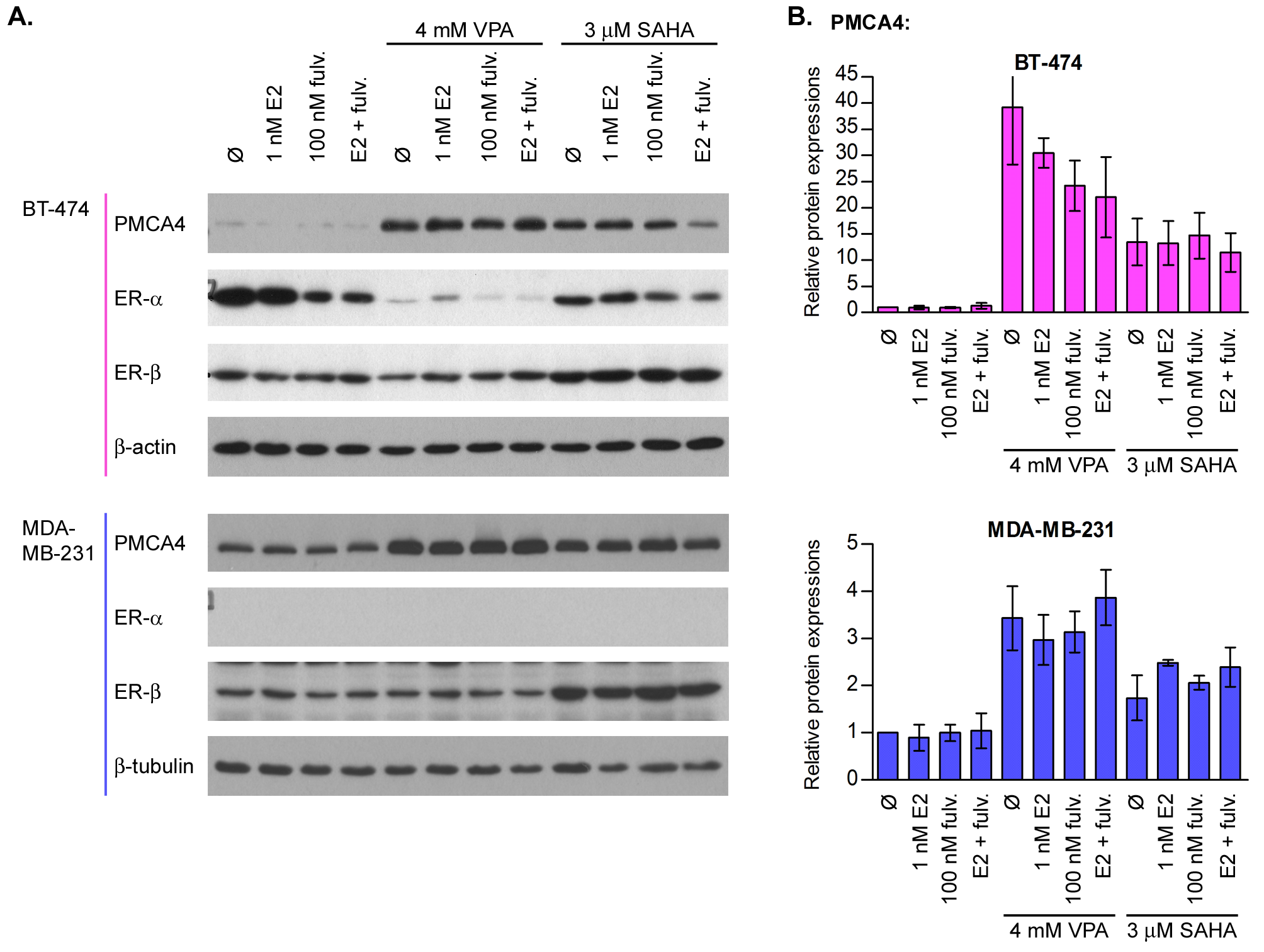

Supplement: Supplementary file 5 — Figure S4. Effects of 17β-estradiol (E2) ± HDAC inhibitor treatments on PMCA4 protein expression in the ER-α positive BT-474 and in the ER-α negative MDA-MB-231 breast cancer cell lines. A: BT-474 and MDA-MB-231 cells were cultured in E2-free culture medium and treated with 1 nM E2 ± 100 nM fulvestrant (fulv.) ± 4 mM VPA or 3 μM SAHA for 4 days as indicated. Equal amounts (30 μg) of total cell lysates were analyzed by Western blotting using the anti-PMCA4 (JA9), anti-ER-α and anti-ER-β antibodies. β-actin served as a loading control. B: Relative PMCA4 protein expression in the examined cell lines. Densitometric values were normalized to the respective β-actin levels and expressed as fold increase over untreated controls. Bars represent mean ± SEM from three independent experiments. (TIF 915 kb) [file 12885_2018_4945_MOESM5_ESM.tif]
